# Null Steering of Adaptive Beamforming Using Linear Constraint Minimum Variance Assisted by Particle Swarm Optimization, Dynamic Mutated Artificial Immune System, and Gravitational Search Algorithm

**DOI:** 10.1155/2014/724639

**Published:** 2014-07-22

**Authors:** Soodabeh Darzi, Tiong Sieh Kiong, Mohammad Tariqul Islam, Mahamod Ismail, Salehin Kibria, Balasem Salem

**Affiliations:** ^1^Department of Electrical, Electronic & Systems Engineering, Universiti Kebangsaan Malaysia, 43600 Bangi, Selangor, Malaysia; ^2^Power Engineering Center & Center of System and Machine Intelligence, College of Engineering, Universiti Tenaga Nasional, 43000 Kajang, Selangor, Malaysia; ^3^Space Science Center (ANGKASA), Universiti Kebangsaan Malaysia, 43600 Bangi, Selangor, Malaysia

## Abstract

Linear constraint minimum variance (LCMV) is one of the adaptive beamforming techniques that is commonly applied to cancel interfering signals and steer or produce a strong beam to the desired signal through its computed weight vectors. However, weights computed by LCMV usually are not able to form the radiation beam towards the target user precisely and not good enough to reduce the interference by placing null at the interference sources. It is difficult to improve and optimize the LCMV beamforming technique through conventional empirical approach. To provide a solution to this problem, artificial intelligence (AI) technique is explored in order to enhance the LCMV beamforming ability. In this paper, particle swarm optimization (PSO), dynamic mutated artificial immune system (DM-AIS), and gravitational search algorithm (GSA) are incorporated into the existing LCMV technique in order to improve the weights of LCMV. The simulation result demonstrates that received signal to interference and noise ratio (SINR) of target user can be significantly improved by the integration of PSO, DM-AIS, and GSA in LCMV through the suppression of interference in undesired direction. Furthermore, the proposed GSA can be applied as a more effective technique in LCMV beamforming optimization as compared to the PSO technique. The algorithms were implemented using Matlab program.

## 1. Introduction

Adaptive beamforming was inaugurated to evolvement in aerospace and military applications with technology firmly fixed on phased-array via the electronically steered antennas [1]. Adaptive antennas were then supposed appropriate to solve the cochannel interference and multipath fading problem for mobile wireless communication. Adaptive antenna array was created in the 1950s by Howells identified as the intermediate frequency side lobe canceller (SLC) [2]. Although SLC technique encompasses the ability of automatic interference nulling, it was not fully adaptive due to fixed pattern for main beam and few controlled elements for ancillary array. This technology simplified the improvement of qualified adaptive array in 1965 by Applebaum. This algorithm was employed to increase the signal-to-noise ratio (SNR) in order to have more efficiency of adaptive antenna. Meanwhile, another adaptive array technique known as linear constraint minimum variance (LCMV) was created by Windrow [3]. This algorithm was developed based on the conventional minimum mean square error (MMSE) for the automatic adjustment of array weights with the privilege of simplicity. Low convergence rate is the main disadvantage of LCMV technique that makes it inappropriate for some applications, while the advantage of it is only that it requires the direction of arrival (DOA) for maximizing the SNR. Formerly, different research works have been presented, which used LCMV for beamforming applications [3–5]. According to the characteristics of LCMV beamforming technique, it has a weak beam pattern and low signal to interference-noise ratio (SINR) value. Solving this problem through conventional empirical approach is very difficult, time consuming, and sometimes in the applied case unmanageable. Consequently, many metaheuristics and exploratory methods have been settled to get the best results for these types of difficulties. Previous studies show that the employment of metaheuristics algorithm has been growing instead of exhaustive and exact procedures. In this regard, approaches such as genetic algorithms (GA) [6, 7], artificial bee colony (ABC) [8, 9], differential evolution (DE) [10], particle swarm optimization (PSO) [11, 12], ant colony optimization (ACO) [13–15], tabu search (TS) [8, 16, 17], artificial immune system (AIS) [18], and clonal selection (CS) [19, 20] have been used to solve a variety of problems in order to improve various issues in antenna system. According to the above-mentioned study, the main goal of this paper is to optimize the LCMV beamforming weights by PSO and gravitational search algorithm (GSA) technique to improve the performance of system in the expect of SINR.

In this investigation, PSO, DM-AIS, and GSA have been applied in uniform linear antenna arrays with 0.5*λ* spacing between adjacent elements at a frequency of 2.3 GHz. The rest of this paper is organized as follows.  Section 2 introduces system model which contains the basics of adaptive beamforming, LCMV technique, and its algorithm. The artificial intelligence (AI) techniques including PSO, DM-AIS, and GSA are summarized in Section 3.  Section 4 shows the application of presented AI in LCMV technique. Simulation results are reported in Section 5, and finally Section 6 concludes this investigation.

## 2. System Model

In this section, the mathematical formulation of the design model for an adaptive beamforming and LCMV technique will be presented in detail.

### 2.1. Adaptive Beamforming

The beamforming technique attempts to make beam toward the signal of interest (SOI) and produce null toward the direction of signals not of interest (SNOI). Signal processing technique automatically adjusts incoming SOIs and SNOIs from collected information and weight. The outputs of the individual sensors were linearly combined after being scaled with the corresponding weights. This process optimizes the antenna array to achieve maximum gain in the direction of the desired signal and nulls in the direction of interferers. For a beamformer, the output at any time *n*, *y*(*n*) is given by a linear combination of the data at *M* antennas, with *x*(*n*) being the input vector, *w*(*n*) the weight vector, and *H* the Hermitian transpose. Consider

(1) $$\begin{array}{c} y\left(n\right)=w^{H}\left(n\right)x\left(n\right). \end{array}$$

Weight vector *w*(*n*) can be defined as follows

(2) $$\begin{array}{c} w\left(n\right)=\overset{M-1}{\underset{N=0}{\sum }}w_{n},\\ x\left(n\right)=\overset{M-1}{\underset{n=0}{\sum }}X_{n}. \end{array}$$

The weight vector at time *n* + 1 for any system that uses the direct gradient vector for upgrading weight vector and avoid the matrix inverse process can be as follows

(3) $$\begin{array}{c} W\left(n+1\right)=W\left(n\right)+\frac{1}{2}\mu \left[\nabla J\left(n\right)\right], \end{array}$$

where *μ* is the step size parameter, which controls the speed of convergence and lies between 0 and 1. While the smallest quantity of *μ* assists the cost function superior estimation and sluggish concurrence, the huge quantity of it may result in a quick union. Nevertheless, the constancy over the minimum value could disappear. Consider

(4) $$\begin{array}{c} 0<\mu <\frac{1}{\lambda }. \end{array}$$

Estimation of gradient vector is written as

(5) $$\begin{array}{c} \nabla J\left(n\right)=-2p\left(n\right)+2R\left(n\right)W\left(n\right),\\ R\left(n\right)=X\left(n\right)X^{H}\left(n\right),\\ P\left(n\right)=d\left(n\right)*X\left(n\right), \end{array}$$

where *R* is the covariance matrix and *p* is the cross-correlation vector.

By integrating (5) into (3), the weight vector can be derived as follows

(6) W(n+1)=W(n)+μ[p(n)−R(n)W(n)]=W(n)+μX(n)[d∗(n)−X(n)W(n)]=W(n)+μXe∗(n).

The following three formulas further define the desired signal as

(7) y(n)=wH(n)x(n),e(n)=d(n)·y(n)W(n+1)=W(n)+μX(n)e∗(n).

### 2.2. Conventional LCMV Beamforming

Numerous algorithms were introduced for the design of an adaptive beamformer [21]. One of the popular approaches for adaptive beamforming was generated by Windrow [3]. His technique leads to an adaptive beamformer with the LCMV. The form of radiation beam in LCMV depends on the knowledge of the received desired signal. Moreover, LCMV technique can estimate the source of interference and control radiation lobe in order to accumulate high power to the desired direction. Although this system does not have to know the radiated power of the desired signal or the direction of interference and white noise, it is capable of suppressing the disturbances as much as possible. The total signal is given by

(8) $$\begin{array}{c} x_{\text{Total}}=x_{s}\left(t\right)+x_{i}\left(t\right)+x_{n}\left(t\right), \end{array}$$

where *x*_*s*_ is the desired signal, *x*_*i*_ is the interference signal, *x*_*n*_ is the noise signal added from Gaussian noise, and *x*_*i*_ + *x*_*n*_ is the undesired signal.

As mentioned in Section 2.1, the array output can be written as follow

(9) $$\begin{array}{c} y=w^{H}x. \end{array}$$

The output power is given by

(10) $$\begin{array}{c} p=\left\{E{\left|y\right|}^{2}\right\}=E\left\{w^{H}xx^{H}w\right\}=w^{H}E\left\{xx^{H}w\right\}=w^{H}R, \end{array}$$

where the *R* covariance matrix should be (*M*, 1) for the received signal *x*.

The LCMV algorithm depends on the steering vector, based on the incident angle that is obtained from the incident wave on each element. The optimum weights are selected to minimize the array output power while maintaining unity gain in the look direction. The LCMV adaptive algorithm is shown in formula (11) when a constant beam pattern is required. Consider

(11) min⁡{wHRxxw}conditionalon⁡wHa(θ0)=g,

where *g* is the complex scalar.

Steering vector *a*(*θ*) is given by [22]

(12) a(θ)=[1exp⁡{j2πλ(sinθi)d}exp⁡{j2πλ(sinθi)(m−1)d}],

where *θ*_*i*_ is the desired angle, *d* is the space between elements of antenna, and *m* is number of elements. All assumptions in this work will be shown later in Sections 4 and 5.

The optimization weight vectors can be calculated by the following formula [22, 23]:

(13) $$\begin{array}{c} W_{\text{LCMV}}=g*\frac{R_{xx}^{-1}a\left(\theta_{0}\right)}{a^{H}\left(\theta_{0}\right)R_{xx}^{-1}a\left(\theta_{0}\right)}. \end{array}$$

That means *M* number of weights as below will be obtained by using *M* number of adaptive antenna elements. Consider

(14) $$\begin{array}{c} w_{\text{MVDR}}=\left[\begin{array}{c} w_{1}\\ w_{2}\\ \cdots \\ w_{M} \end{array}\right]. \end{array}$$

The total noise, containing uncorrelated noise and interferences, is decreased by the minimization procedure. Notably, continually sustaining the output signal, the minimization of the total interference and noise is the same as maximizing the output SINR. The number of interference sources should be equal to or less than *M* − 2 since an array with *M* elements has only *M* − 1 degrees of freedom and has been employed by the constraint in the look direction for cancelling interferences in order to maximize the SINR. The LCMV algorithm is unsuitable when the users spread in all directions. This is called a multipath environment due to capability of incresing sensitivity in only one direction [24]. The multipath interference occurs where numeorus scatterers neighbor in the same base station and users are in populated urban regions. Consequantly, LCMV beamforming technique may have an inappropriately low null level, in the situation of undesired interference signals which may have significant effect on performance of the system.

## 3. Methodology

In this section, the PSO, DM-AIS, and GSA are summarized as a basis to describe the proposed model which is used to solve adaptive beamforming problems in LCMV technique.

### 3.1. Particle Swarm Optimization (PSO)

Particle swarm optimization (PSO) is a heuristic robust stochastic optimization technique that was introduced by Kennedy and Eberhart [25]. This method is inspired from the simulation of the social behaviors of animals (flock of birds). PSO updates the population of particles by adding an operator based on the fitness information achieved from the environment. Therefore, the individuals of the population would be estimated to move to the improved solution. The position and velocity of every population member are updated via the following formula [26]:

(15) vid(t+1)=wvid(t)+c1R1(pbestid(t)−pid(t))+c2R2(gbest(t)d−pid(t)),

(16) $$\begin{array}{c} p_{i}^{d}\left(t+1\right)=p_{i}^{d}\left(t\right)+v_{i}^{d}\left(t+1\right). \end{array}$$

In the mentioned formula, velocity is *v*_*i*_^*d*^ with dimension *d* and particle *i*, *p*_*i*_^*d*^ shows the position of particle *i*, *t* is the iteration number, and *d* represents the dimension of search space. *R*_1_ and *R*_2_ are uniform random variables as the random cognitive coefficient and random social coefficient, respectively, that are applied to make a randomized characteristic in the search region. *w* is the inertia weight to balance the local and global search abilities of the particles. *c*_1_ and *c*_2_ are cognitive and social coefficient, *p*best is the individual best solution for *i*th particle, and *g*best represents the global best solution for the population. The positions are updated according to formula (16) and their fitnesses are evaluated. This process is repeated untill a termination criterion is met. The work in [27] has suggested six different steps for solving the problem by using the PSO as below.

**Step 1 .**

Generate randomly the particles and their velocity in the dimensional search space.

**Step 2 .**

Evaluate the fitness function for each one.

**Step 3 .**

Update *p*best for each position; if the *p*best value is lower than current fitness value of the particle, replace it by current value.

**Step 4 .**

Update *g*best; if the *g*best value is lower than current fitness value of the particle, replace it by current value.

**Step 5 .**

Update the velocity and position of each particle using (15) and (16), respectively.

**Step 6 .**

Repeat Steps 2–5, until the maximum number of iteration is met. The explanations on how PSO is used to optimize the weights of LCMV technique is given in Section 4.

### 3.2. Dynamic Mutated Artificial Immune System (DM-AIS)

One of the metaheuristic algorithms is artificial immune systems (AIS) [28, 29] with significant popularity in the field of optimization. AIS is mimicking the behavior of human immune system towards foreign elements in a host body. The human immune system makes active antibodies when antigens attack the body. In addition, the human immune system produces great amount of antibodies that fix powerfully to a specific antigen through its cloning process. The mutation rate of cloned antibodies is inversely proportionate to the affinity of antigens. Thus, the lowest affinity antibodies will result in the highest mutation rates. The general steps of AIS are described as below.

- Initialize of random solutions (antibodies).
- Calculate fitness for each solution.
- Store the best fitness value and its associated solution.
- Select a subset of antibodies for cloning.
- Apply mutation for each cloned antibody.
- Calculate fitness for each cloned antibody after mutation.
- Compare new fitness with best fitness value.
- If fitness of cloned antibody better than previous fitness, replace cloned antibody as antibody.
- Repeat until termination criteria are met.

DM-AIS [18] is a type of AIS algorithm with a new dynamic mutation function. The new population of antibodies is derived from the fitness value based on dynamic mutation rate. The new dynamic mutation rate is then able to improve the convergence rate of the antibody solution.

### 3.3. Gravitational Search Algorithm (GSA)

One of the latest search algorithms for heuristic population is gravitational search algorithm (GSA). GSA is employed as an artificial world of masses following the gravitation of Newtonian laws [30]. All the GSA search agents (individuals) can be considered as objects and their masses are the factors of evaluate them.

Gravity force is the cause of the movement of all objects globally toward the objects with heavier masses. It means that optimum solutions of the problems are represented by the heavy masses. In this method, the new position and velocity of agent *i* will be upgraded based on the formula below:

(17) vid(t+1)=randi×vid(t)+aid(t),pid(t+1)=pid(t)+vid(t+1).

In the mentioned formula, velocity of *i*th agent is *v*_*i*_^*d*^ in dimension *d*, *p*_*i*_^*d*^ is position of *i*th agent at iteration number *t*. rand_*i*_ represents a random variable in order to provide a randomized characteristic for the search space as well as enhancement of the finding the global optimal chance, *a*_*i*_^*d*^ is the acceleration of agent *i* in dimension *d*, and could be obtained as below:

(18) aid(t)=∑j∈kbest,j≠irandiG(t)Mj(t)Ri,j(t)+ε(pjd(t)−pid(t)),

where *a*_*i*_^*d*^ is the acceleration of *i*th agent in dimension *d* and rand_*j*_ is random value; according to formula (19), the gravitational constant at time *t* is *G*(*t*); *M*_*j*_ is mass of *j*th agent represented in formula (20); *ε* is a minor element to prevent division by zero and the *R*_*i*,*j*_(*t*) is the Euclidean distance that is represented as *R*_*i*,*j*_(*t*) = ||*p*_*i*_(*t*),*p*_*j*_(*t*)||_2_. It is valued to reference that we employ *R* as a replacement of *R* ^2^ in formula (18) due to the tests offered in [30] which illustrate that *R* offers better output in comparison with *R* ^2^. *k*best is a control function that is able to advance the performance of GSA. This function controls the exploitation and exploration with initialization to *k*_0_ at the starting and reducing with each iteration [30]. *k*_0_ is adjusted to *N* (overall amount of agents) and is reduced to 1 linearly. In formula (18), the gravitational constant *G*(*t*) is a reducing function of time when it is set to *G*_0_ at the starting and will be decreased exponentially as shown in formula below:

(19) $$\begin{array}{c} G\left(t\right)=G_{0}\times \exp\left(-\beta \times \frac{t}{t_{\max}}\right). \end{array}$$

In formula (19), *β* is a gradient constant value, *t* is the current repetition, and *t*_max⁡_ is the maximum repetition number. Furthermore, the mass of agents in formula (18) is examined with employing the formula (20) as a below:

(20) $$\begin{array}{c} M_{i}\left(t\right)=\frac{m_{i}\left(t\right)}{\sum_{j=1}^{N}m_{j}\left(t\right)}, \end{array}$$

in which

(21) $$\begin{array}{c} m_{i}\left(t\right)=\frac{\text{fi}{\text{t}}_{i}\left(t\right)-\text{worst}\left(t\right)}{\text{best}\left(t\right)-\text{worst}\left(t\right)}. \end{array}$$

In formula (21), fit_*i*_(*t*) is the fitness value of the agent *i* at time *t*. worst(*t*) and best(*t*) are the worst and best fitness of all agents, respectively.

## 4. Proposed LCMV Beamforming Assisted by Gravitational Search Algorithm, Dynamic Mutated Artificial Immune System, and Particle Swarm Optimization

The PSO, DM-AIS, and GSA were utilized to enhance the SINR value of the LCMV beamforming technique in this paper. The smart antenna will try to optimize via PSO, DM-AIS, and GSA iteration process to make deep null at the interference sources in order to get the maximum SINR.

In these algorithms, the *w* (weight vector) will be used as the system population. These algorithms will initiate by generating the *N* particles, which is indicated by *W*_*N*_ weight vectors. Moreover, the number of produced weight vectors is dependent on the population size *P*_size_. For the first generation, the first set of weight vectors *W*_1_ is obtained from the computation of the conventional LCMV weight vector. The weight vectors in every particle contain an *M* number of weight vectors, depending on the antenna elements usedor the number of sensors. The weight vectors in the population of any iteration can be illustrated in matrix format as a below:

(22) WNM=[Wlcmv1Wlcmv2⋯WlcmvMW11W12⋯W1MW21W22⋯W2M········Wn1Wn2⋯WnM],

where *W*_*NM*_ are the weight vectors of total population *N* with *M* sensors in each antenna and *W*_lcmv_ are the weight vectors from LCMV beamformer.

PSO, DM-AIS, and GSA: *p*_*i*_^*d*^ = *W*_*n*_^*M*^, where *d* = *M*, *i* = *n*, and *i*th Particle at *d*th dimension can be presented as *n*th weight at dimension *M*.

Each set of particles *W* has amplitude and phase (*A*∀ *θ*) to steer the radiation beam toward its target user and place the deep null toward the interference sources to achieve the optimum SINR. The best weight vector is determined according to the fitness value obtained from fitness function as shown below. Consider

(23) $$\begin{array}{c} \text{Fitness\_Function}\left(\text{FF}\right)=\frac{P_{\text{User}}}{\sum_{n=1}^{N}P_{\text{Inter\_}n}+\text{Noise}}, \end{array}$$

where *P*_User_ is the power of target user, *P*_Inter_ is the power of interference, and *n* is the number of interference sources.

Table 1 shows the used parameters of PSO and GSA in this study. The parameters of GSA are chosen according to the guidelines and recommendations presented in [30]. These configurations of GSA have also been utilized extensively after the development of GSA [31–34]. The linearly decreasing inertia coefficient of PSO was chosen to allow initial exploration while not degrading the convergence rate. The cognitive and social coefficients were chosen based on the recommendations in the literature [35, 36]. The maximum iterations for DM-AIS are 500 for fair comparison with GSA and PSO. DM-AIS parameters implemented in this study were the same as in [18].

## 5. Experimental Results and Discussion

The effectiveness of the proposed PSO, DM-AIS, and GSA techniques in LCMV in comparison with the conventional LCMV beamforming is investigated by considering four cases of different interferences and elements in this section. All cases have one user at 0°, while the number of interferences and elements changes in each case. The first case is interference signal at 20° by using 4-element patch antenna; the second case is two interference signals at 20° and 60° by using 4-element patch antenna. Third and fourth cases include same interference at cases 1 and 2 but using 8-element patch antenna. Optimization result of PSO and GSA are obtained from 10 cycle's simulation and the best results are plotted.

### 5.1. Case 1: One User, One Interference, Four Elements

**Figure 1:**
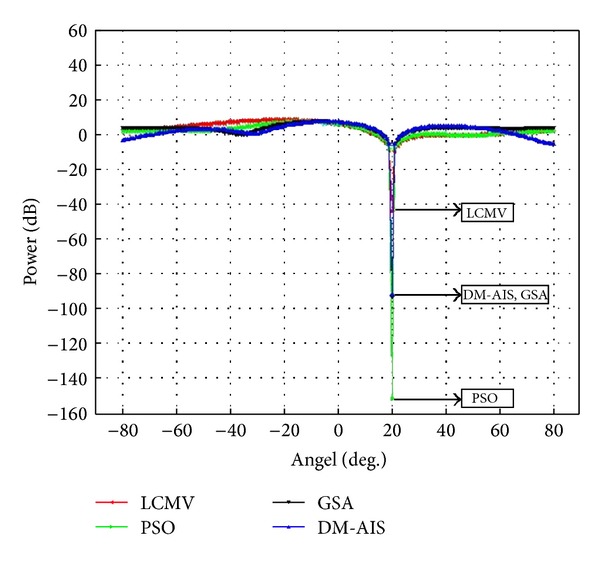
Comparison of performance of power response LCMV, PSO-LCMV, DM-AIS-LCMV, and GSA-LCMV for user at 0° with interference at 20° by using 4 elements.

One interference source at 20° and user at 0° by using 4-element patch antenna has been assumed in the first case study. The simulation result is shown in Figure 1.

Table 2 compares the power values of the above-mentioned four methods. It is obvious that the GSA method performs better than PSO and DM-AIS with the same number of iterations. It means GSA is superior as compared to PSO and DM-AIS because it creates deeper null in the interference direction while increasing power at desired direction. This result also demonstrates that the improvements of SINR are 52.01%, 53.63%, and 54.14%, respectively, by PSO, DM-AIS, and GSA as compared to conventional LCMV method.

### 5.2. Case 2: One User, Two Interferences, Four Elements

**Figure 2:**
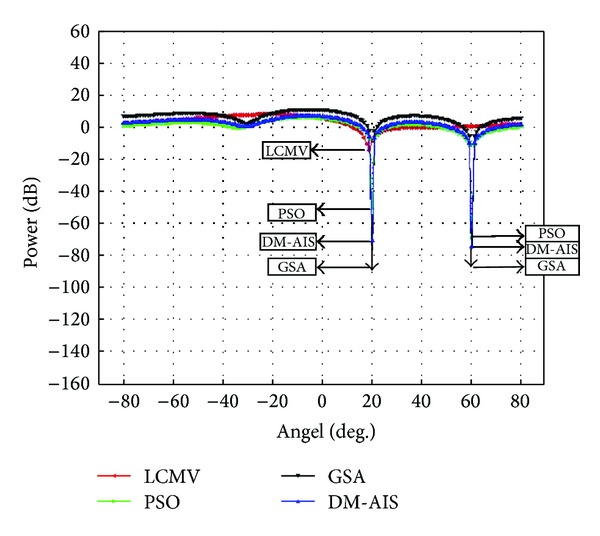
Comparison of performance of power response LCMV, PSO-LCMV, DM-AIS-LCMV, and GSA-LCMV for user at 0° with interference at 20° and 60° by using 4 elements.

Two interference sources at 20°, 60° by using 4-element patch antenna and user at 0° has been assumed in the second case study. The simulation outcome is displayed in Figure 2.

Table 3 compares the power values and SINR of the methods mentioned previously. The GSA method achieves better performance with the same iterations in Case 2 also. This shows that the proposed methods can achieve better nulls than conventional LCMV for two interference cases. In addition, this table presents the ratio of SINR 4.40 dB through conventional LCMV while the PSO, DM-AIS, and GSA improve these values 1176.81%, 1556.59%, and 1732.95%, respectively.

### 5.3. Case 3: One User, One Interference, Eight Elements

One interference at 20° by using 8-element patch antenna and user at 0° has been assumed in the third case study.

**Figure 3:**
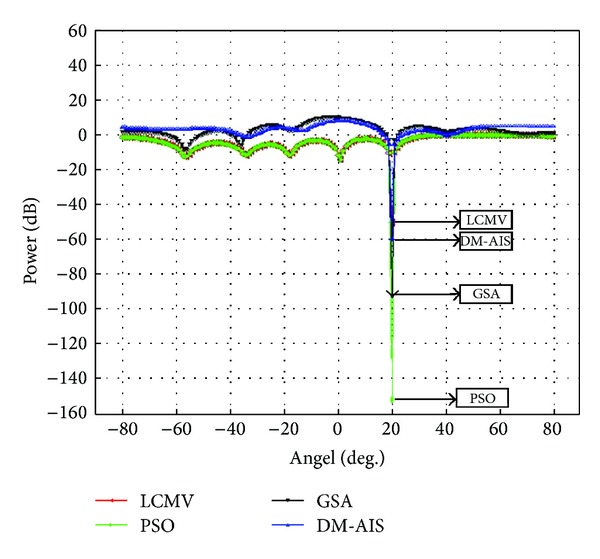
Comparison of performance of power response LCMV, PSO-LCMV, DM-AIS-LCMV, and GSA- LCMV for user at 0° with interference at 20° by using 8 elements.

The results of Case 3 are consistent with the findings of previous two cases. GSA-LCMV outperforms conventional LCMV, DM-AIS-LCMV, and PSO-LCMV significantly for eight-element array also, as illustrated in Figure 3. Thus, the superiority of GSA over other techniques is independent of number of array elements.  Table 4 demonstrates the ratio of SINR 38.93 dB through conventional LCMV, while the PSO, DM-AIS, and GSA improve these values 49.73%, 72.61%, and 105.26%, respectively.

### 5.4. Case 4: One User, Two Interferences, and Eight Elements

Two interference sources at 20°, 60° by using 8-element patch antenna and user at 0° have been assumed in the fourth case study.

**Figure 4:**
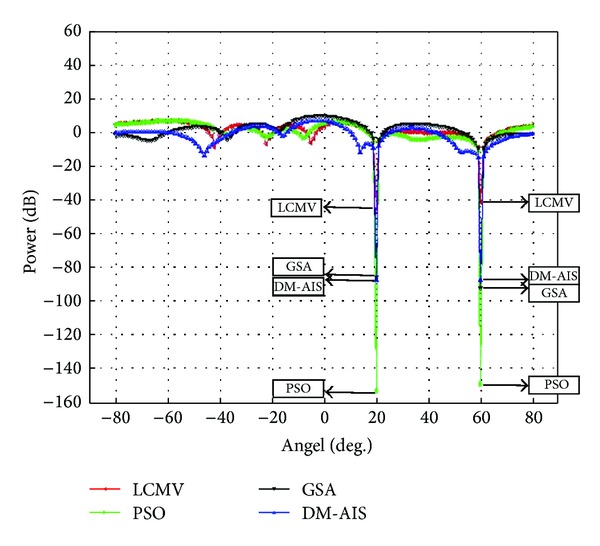
Comparison of performance of power response LCMV, PSO-LCMV, DM-AIS-LCMV, and GSA-LCMV for user at 0° with interference at 20°, 60° by using 8 elements.

Case 4 confirms the findings of Case 3 in terms of superiority of GSA, as shown in Figure 4.  Table 5 shows that GSA-LCMV achieves highest power in desired direction and lower power at nulls than conventional LCMV. PSO is known to have premature convergence issues. Thus, GSA is able to consistently outperform PSO in array optimization problems. Besides, this result also illustrates that the development of SINR is 72.62%, 75.50%, and 82.22%, respectively, by PSO, DM-AIS, and GSA as compared to conventional LCMV technique. The DM-AIS was developed to improve the performance of AIS for beamforming applications [18]. Based on the results presented in this section, it converges to significantly better solutions than PSO consistently. However, GSA still outperforms DM-AIS with superior SINR in all scenarios.

## 6. Conclusion

This paper applied PSO, DM-AIS, and GSA in linear antenna arrays with different number of elements to control the nulling of interference and the directionality towards the desired signal. The results of the LCMV assisted by proposed approaches were compared with conventional LCMV, and the effectiveness of the proposed approaches in minimizing the power of interference and increasing SINR was observed. The result of LCMV beamforming assisted by GSA algorithm is better than PSO and DM-AIS algorithm and also its conventional LCMV beamforming algorithm. This new proposed GSA-LCMV can be useful for smart antenna for the radiation pattern synthesis because of its good accuracy and not requiring complicated mathematical functions.

## Figures and Tables

**Table 1:** Used parameters of PSO and GSA briefly in this study.

Parameter	Description	PSO	GSA
*N *	Population size	10	10
*d *	Dimension of the search space	4,8	4,8
*t*_max⁡_	Maximum iteration	500	500
*W *	Weight	Start weight = 0.9 End weight = 0.4	N.A
*c*_1_	Cognitive coefficient	2	N.A
*R*_1_	Random cognitive coefficient	Rand(0, 1)	N.A
*c*_2_	Social coefficient	2	N.A
*R*_2_	Random social coefficient	Rand(0, 1)	N.A
*G*_0_	Initial value of gravitational constant	N.A	100
*β*	Gradient constant	N.A	20
*ε*	Zero offset constant	N.A	2.22*e − *16

**Table 2:** Comparison of weight vectors, power values, and SINR calculation for conventional LCMV, PSO-LCMV, DM-AIS-LCMV, and GSA-LCMV for user at 0° with interference at 20° by using 4 elements.

Algorithm	Weights	Power at 0° (W)	Power at 20° (W)	SINR (dB)
LCMV	−0.0034 − 0.9316*i* −2.1723 − 1.8276*i* −2.1723 + 1.827*i* −0.0034 + 0.9316*i *	4.35	4.07 × 10^−5^	50.27

PSO	−1.0592 − 0.3784*i* −1.1478 − 0.3784*i* −1.8144 + 1.27657*i* 0.0176 + 1.2784*i *	4.38	1 × 10^−15^	76.42

DM-AIS	1.2962 + 0.9929*i* 0.3293 + 0.9559*i* 1.2026 + 0.3361*i* 1.9141 + 0.1246*i *	5.31	6.29 × 10^−10^	77.23

GSA	0.6579 + 0.8473*i* 1.3924 + 1.1853*i* 0.6916 + 0.5625*i* 2.5054 − 0.4960*i *	5.65	5.03 × 10^−10^	77.50

**Table 3:** Comparison of weight vectors, power values, and SINR calculation for conventional LCMV, PSO-LCMV, DM-AIS-LCMV, and GSA-LCMV for user at 0° with interference at 20° and 60° by using 4 elements.

Algorithm	Weights	Power at 0° (W)	Power at 20° (W)	Power at 60° (W)	SINR (dB)
LCMV	0.0067 − 0.7860*i* −1.7890 − 1.6179*i* −1.7888 + 1.6184*i* 0.0069 + 0.7860*i *	3.56	1.5 × 10^−1^	1.14 × 10^0^	4.40

PSO	0.2620 + 1.0535*i* 0.1924 + 0.6244*i* 0.5209 + 0.8704*i* 1.3205 + 0.3789*i *	3.72	8.69 × 10^−6^	1.54 × 10^−7^	56.18

DM-AIS	0.7471 + 1.2004*i* 0.4117 + 0.5250*i* 0.7398 + 0.8941*i* 1.7452 + 0.1232*i *	4.56	9.44 × 10^−8^	3.96 × 10^−8^	72.89

GSA	−4.1832 − 1.6647*i* −2.5456 + 0.9557*i* −1.8412 + 0.4129*i* −3.3839 + 2.0752*i *	12.08	1.78 × 10^−9^	2.18 × 10^−9^	80.65

**Table 4:** Comparison of weight vectors, power values, and SINR calculation for conventional LCMV, PSO-LCMV, DM-AIS-LCMV, and GSA-LCMV for user at 0° with interference at 20° by using 8 elements.

Algorithm	Weights	Power at 0° (W)	Power at 20° (W)	SINR (dB)
LCMV	0.0317 + 0.1445*i* 0.1194 + 0.0909*i* −0.2264 + 0.1311*i* 0.0990 − 0.2212*i* 0.1017 + 0.2525*i* −0.1896 − 0.12725*i* 0.1060 − 0.1115*i* 0.0227 − 0.1350*i *	6.90 × 10^−2^	8.71 × 10^−6^	38.93

PSO	0.0317 + 0.1445*i* 0.1194 + 0.0909*i* −0.2263 + 0.1311*i* 0.0990 − 0.2212*i* 0.1017 + 0.2525*i* −0.1897 − 0.1271*i* 0.1061 − 0.1115*i* 0.0227 − 0.1349*i *	6.92 × 10^−2^	2.23 × 10^−9^	58.30

DM-AIS	−0.0864 + 0.7410*i* 0.9922 + 0.1975*i* 1.6119 + 0.4745*i* 0.1607 + 0.5461*i* 1.3573 + 0.2932*i* −0.1193 + 0.6657*i* 0.9244 + 0.7284*i* 0.0049 + 0.5399*i *	6.40 × 10^0^	1.11 × 10^−6^	67.20

GSA	−0.6737 + 0.8571*i* 0.6194 − 0.0643*i* 1.3861 + 0.6505*i* 0.8473 + 0.8198*i* 1.1431 + 0.9980*i* 1.5379 + 1.0542*i* 1.8363 + 0.9692*i* 1.3944 + 0.42148*i *	9.90 × 10^0^	6.30 × 10^−10^	79.92

**Table 5:** Comparison of weight vectors, power values, and SINR calculation for conventional LCMV, PSO-LCMV, DM-AIS-LCMV, and GSA-LCMV for user at 0° with interference at 20°, 60° by using 8 elements.

Algorithm	Weights	Power at 0° (W)	Power at 20° (W)	Power at 60° (W)	SINR (dB)
LCMV	−0.6890 + 0.4136*i* 0.2415 + 1.1050*i* −0.6307 − 0.3236*i* −0.2050 + 0.4525*i* −0.1819 − 0.4779*i* −0.5573 + 0.2462*i* 0.2360 − 1.2187*i* −0.7214 − 0.4358*i *	2.51	2.98 × 10^−5^	7.66 × 10^−5^	43.73

PSO	−0.6004 + 0.5241*i* 0.2293 + 1.1021*i* −0.5799 − 0.3262*i* −0.5170 + 0.9217*i* −0.1831 − 0.3784*i* −1.0433 + 0.2464*i* 0.0982 − 0.3784*i* −0.6887 − 0.3784*i *	3.54	1 × 10^−15^	1 × 10^−15^	75.49

DM-AIS	−0.4890 + 0.5137*i* −0.2718 + 0.6251*i* 0.0371 + 0.4669*i* 0.2083 + 0.6415*i* −0.6586 + 0.7074*i* −0.1554 + 0.7897*i* 0.3603 + 0.5786*i* 0.3198 + 0.5346*i *	4.90	1.70 × 10^−9^	1.79 × 10^−9^	76.75

GSA	−0.3417 + 0.1177*i* 0.1586 + 1.0271*i* 1.1506 + 0.3389*i* 1.8499 + 0.6928*i* 1.5246 + 1.0174*i* 0.7917 + 0.3530*i* 1.5273 + 0.7368*i* 1.4564 + 0.8934*i *	9.62	2.64 × 10^−9^	6.36 × 10^−10^	79.69
